# Interactive gamified health education to improve knowledge, attitudes, and practices on intestinal parasitic infections: A community pilot in Malaysia’s urban public housing

**DOI:** 10.1371/journal.pntd.0014509

**Published:** 2026-07-15

**Authors:** Nabila AbuBakar, Beh Jia Ni, Suresh V. Kuchipudi, Siti Nursheena Mohd Zain

**Affiliations:** 1 Institute of Biological Sciences, Faculty of Science, Universiti Malaya, Kuala Lumpur, Malaysia; 2 Department of Infectious Diseases and Microbiology, School of Public Health, University of Pittsburgh, Pittsburgh, Pennsylvania, United States of America; London School of Hygiene and Tropical Medicine, UNITED KINGDOM OF GREAT BRITAIN AND NORTHERN IRELAND

## Abstract

Intestinal parasitic infections (IPIs) remain a major yet under-recognised public health challenge in rapidly urbanising settings. In Malaysia, most IPIs research has focused on indigenous communities, leaving critical knowledge gaps among urban poor populations living in public housing programmes (PHPs), where inadequate sanitation and waste management can sustain disease transmission. We conducted a pre–post pilot at one PHP in the Klang Valley to evaluate a multi-component community-based health education intervention incorporating gamified participatory learning activities on IPI-related knowledge, attitudes, and practices (KAP). Ninety participants from three age groups: children (7–12 years), adolescents (13–17 years), and adults (>18 years), completed standardised KAP questionnaires immediately before and after the intervention. The programme combined educational briefings, illustrated materials, and age-tailored interactive activities (e.g., card games, charades, Pictionary, Bingo, and matching activities) to deliver key prevention and hygiene messages. At baseline, adolescents scored highest across KAP domains, followed by children and adults. Post-intervention, mean scores improved significantly in all domains: knowledge improved by 60.5 ± 24.7 points, attitudes by 26.9 ± 32.5 points, and practices by 29.3 ± 33.4 points (p < 0.001). Gains were observed across age groups, suggesting broad acceptability and learning effectiveness. This participatory and culturally attuned approach demonstrates potential as a low-cost, scalable strategy to strengthen health literacy in underserved urban communities. As the social and environmental drivers of IPIs are common in many rapidly growing cities, this approach is generalisable and aligns well with One Health efforts to address enteric infections at the human–animal–environment interface. This low-cost, scalable approach can complement deworming, sanitation, and surveillance programmes by translating prevention messages into actionable behaviours.

## Author summary

Intestinal parasitic infections are often thought of as a health problem of the past, but in many marginalised communities, including the urban poor, this issue quietly persists. Most research in Malaysia has focused on rural and indigenous communities, yet urban poor populations remain equally vulnerable. Overcrowding, poor waste management, and limited health awareness put this often-overlooked community at ongoing risk. Hence, in this study, we asked a simple question: can learning about intestinal parasites be made fun? Instead of the usual conventional health talks, we introduced games and an interactive health education intervention. Age-appropriate participatory learning activities, including adapted games such as Pictionary, Bingo, and matching card games, were combined with educational briefings and illustrated materials to deliver key hygiene and prevention messages. Participants completed questionnaires before and after the intervention, assessing knowledge, attitudes, and practices related to intestinal parasitic infections. Correct knowledge responses were scored numerically, while attitudes and practices were assessed using simple rating scales, with higher scores indicating better knowledge, awareness, and preventive behaviours. Post-intervention findings showed marked improvements across all age groups, suggesting that culturally relevant and engaging community-based education can strengthen health literacy in underserved urban settings.

## Introduction

Intestinal parasitic infections (IPIs) remain a public health concern, particularly among low-income populations living in densely populated urban housing. In Malaysia, the People’s Housing Programme (PHPs) was initiated by the Ministry of Housing and Local Government [1] to offer affordable housing to low-income groups and reduce urban slums. Rapid urbanisation, high population density, and limited living space often result in overcrowding in housing schemes. Furthermore, inadequate waste disposal practices, poor maintenance of communal areas, and indiscriminate waste management, including overflowing waste collection areas and breeding grounds for pests, can create environments that facilitate the persistence and transmission of intestinal parasites [2,3]. A study conducted by Sahimin et al. revealed a 22.5% parasitic infection rate among residents of these public housing programmes, underscoring the susceptibility of these communities to communicable diseases [4].

IPIs are primarily transmitted through inadequate sanitation and hygiene practices. While once associated predominantly with rural areas, these infections are now increasingly observed in urban settings. Common parasites such as *Ascaris lumbricoides* (roundworm), *Trichuris trichiura* (whipworm), and *Ancylostoma duodenale* (hookworm) can lead to malnutrition, anaemia, stunting, and even cognitive impairments, particularly in children [5,6]. Despite the known health consequences, IPIs remain under-addressed in Malaysia, with no national policy specifically targeting their prevention and control. Existing programmes, such as the National Environmental Sanitation programme, focus mainly on hygiene awareness and deworming. However, the programme’s effectiveness in reducing the burden of soil-transmitted helminths and other IPIs remains uncertain [5].

Globally, IPIs disproportionately impact developing regions, especially in tropical and subtropical climates. Hookworm, ascariasis, trichuriasis, and amebiasis rank among the top ten most common infections worldwide. The World Health Organization (WHO) estimates that ascariasis causes about 4,300 deaths each year, hookworm about 45,000, and amoebiasis approximately 54,000 [7]. In some sub-Saharan and Middle Eastern countries, prevalence rates among schoolchildren range from 50% to 90% [8], highlighting the ongoing global burden and the need for effective and sustainable interventions. While Malaysia has seen a national reduction in IPI prevalence from 52.4% (1970s) to just 1.0% (2012), this advancement may be slipping back in urban poor communities, such as those in the PHP, which remain disproportionately affected [4].

Socioeconomic and environmental factors such as overcrowding, inadequate waste disposal, and unhygienic practices increase the risk of IPIs [4,9]. Historical evidence supports the persistent presence of these infections in urban squatter settlements and flat-dwelling populations, even amidst national improvements [10]. In rapidly urbanising contexts, structural barriers (e.g., inconsistent water, sanitation, and hygiene services; limited health literacy) can undermine the uptake of deworming and hygiene interventions, reinforcing inequities in infection risk.

Assessing community knowledge, attitudes, and practices (KAP) is essential for identifying behavioural and informational gaps that may sustain the transmission of IPIs. KAP surveys can reveal insights into misconceptions, hygiene behaviours, and preventive practices, thereby offering a foundation for targeted health interventions [11]. However, conventional KAP assessments often rely on lengthy questionnaires and didactic health communication approaches, which may reduce participants’ interest, attention, and willingness to engage over time [11]. In underserved communities, barriers such as health literacy, survey fatigue, and reduced familiarity with formal health research may further hinder participation and response quality [12].

Sociodemographic factors such as age, educational level, and gender further shape vulnerability to IPIs. Children, particularly those aged 5–14, are consistently reported as the most affected group due to their developing immune systems, exposure through play in contaminated environments, and limited awareness of hygiene practices [9]. Zia et al. [10] and Kassaw et al. [13] demonstrated that less than half of parents and caregivers possess adequate knowledge of IPIs prevention in endemic areas.

Gamification, the integration of game-based elements in non-game settings, has become a promising tool in health education to enhance engagement, improve learning outcomes, and behavioural change [14]. By incorporating gamified features such as interactive quizzes, challenges, and rewards, gamified interventions can make learning more enjoyable and effective, particularly among younger audiences and demographically diverse communities. Yet evidence for gamified IPIs education in urban Southeast Asian settings remains limited, and the scalability and short-term impact on KAP in high-density housing warrant empirical evaluation.

This pilot study explores gamification as a tool to assess and improve knowledge, attitudes, and practices (KAP) related to intestinal parasitic infections among residents of People’s Housing Programme (PHPs) in the Klang Valley, Malaysia. By evaluating the effectiveness of gamification in enhancing IPIs awareness and promoting healthier behaviours, this study hopes to contribute to more effective public health strategies for low-income urban populations. These conditions are not unique to Malaysia; similar patterns of crowding, inadequate sanitation, and environmental contamination are seen in rapidly growing cities around the world, such as Mumbai, Nairobi, and Brazil, where dense living conditions may contribute to the persistence and transmission of infectious diseases, including IPIs [15–17]. Hence, looking at IPIs through a One Health lens underscores how human behaviour, the local environment, and contact with animals and contaminated soil or water come together to sustain transmission. Findings from this urban public housing setting are relevant to other low-income, high-density communities worldwide and can inform simple, community-based interventions that span human, animal, and environmental health.

## Methodology

### Ethics statement

This study was approved by the Universiti Malaya Research Ethics Committee (UMREC) (UM.TNC2/UMREC_1162) and conducted following its guidelines. All participants provided written, signed consent forms, and parental or legal guardian consent was obtained for children prior to data collection.

### Study area, sample size, and participant recruitment

This study was conducted at a People’s Housing Programme (PHP) in the Bukit Bintang district (3.1388° N, 101.7059° E) of Klang Valley, Malaysia. The study involved 90 participants: 30 children (7 – 12 years), 30 adolescents (13 – 17 years), and 30 adults (> 18 years). The participants were recruited through community-based convenience sampling, in collaboration with local community leaders and residents’ association, based on availability and willingness to participate.

As this was a pilot study, no formal sample size calculation was performed. Instead, sample size was determined pragmatically based on feasibility, available resources, and expected recruitment capacity within the study site. Pilot studies are primarily designed to test study procedures, resolve logistical challenges, and assess initial effects rather than produce statistically generalised results [18]. Smaller sample sizes are often sufficient in pilot studies [19] to inform the development of larger interventions, particularly in community settings where resources and participant availability are limited [18,19]. Pilot and feasibility studies are therefore not intended for hypothesis testing but rather for refining methodology and informing future large-scale studies. Stratification by age further enables preliminary comparisons across various life stages, providing valuable insights for targeted future interventions.

Recruitment was done in close collaboration with the local residents’ association, which played a key role in disseminating information about the study and encouraging community involvement. The inclusion criteria required the participants to (i) be residents of the said PHP, (ii) fit into the lower-income household [<RM5,249 (US$1 = RM 4.68)], and (iii) complete both the pre- and post-KAP surveys as well as participate in all scheduled educational activities.

### Study procedure

The study was conducted over three half-day sessions, each dedicated to a different age group. All sessions followed a structured, multi-component health education intervention that incorporated gamified and participatory learning activities to improve participants’ knowledge, attitudes, and practices (KAP) regarding intestinal parasitic infections (IPIs).

Participants first completed a baseline KAP assessment prior to any educational input, which was crucial for measuring their pre-existing KAP. This was followed by an age-appropriate educational briefing that covered the transmission, symptoms, prevention, and treatment of IPIs. To enhance retention, children were supplemented with comic booklets, while adolescents and adults received illustrated brochures with key messages about IPIs.

Participants then rotated through five interactive learning stations, each featuring games adapted from commonly known activities, including Simon Says, Charades, Bingo, Musical Chairs, and Happy Families. These games were specifically adapted to promote health messages about IPIs. They were also intentionally designed to be offline, ensuring inclusivity, as many community members had limited digital access.

To sustain motivation and promote active participation, small incentives such as stickers, enamel pins, and collectible cards were given to participants who answered correctly during the activities. After completing all stations, participants proceeded to complete the post-intervention KAP survey. Anthelmintic treatment was provided at the end of the programme, together with administration instructions as part of routine public health support.

### Knowledge, Attitudes, and Practices (KAP) questionnaire

The primary data collection instrument was a structured pre- and post-survey, comprising three sections: knowledge (11 items), attitudes (10 items), and practices (11 items), along with sociodemographic data. Knowledge items were scored dichotomously (1 = correct, 0 = incorrect or ‘not sure’). Attitude and practice items were evaluated using 3-point Likert scales. Composite domain scores were generated for knowledge, attitudes, and practices by summing individual item scores. Higher scores indicated better knowledge, more positive attitudes, and improved preventive practices. These continuous scores were used for all statistical analyses. The questionnaire was previously validated for content and face validity, and piloted among residents of PHP communities to ensure clarity, relevance to the context, and suitability before this project began.

### Data analysis

Data were entered into Microsoft Excel for cleaning and cross-checked by two independent researchers. The final dataset was analysed using the Statistical Package for the Social Sciences (SPSS) version 27.0. Continuous variables were summarised as mean (SD), and categorical variables as frequencies and percentages. Normality was assessed using skewness and kurtosis. An independent t-test was used to compare continuous variables between two groups, and a one-way ANOVA was used to compare continuous variables among more than two groups. Post hoc tests were conducted using Student-Newman-Keuls, Tukey HSD, Tukey B, and Scheffé. A paired-sample t-test was employed to assess pre- and post-intervention differences.

## Results

We first describe participant characteristics, then summarize baseline KAP distributions, compare groups by gender and age, and finally report pre–post changes following the intervention (with effect sizes and 95% CIs where applicable).

### Sociodemographic profile of participants

A total of 90 participants were successfully recruited for this study, comprising 32 males (35.56%) and 58 females (64.44%). The participants were evenly distributed across three age groups: 30 children aged 7–12 years (33.33%), 30 adolescents aged 13–17 years (33.33%), and 30 adults aged 18 and above (33.33%). All participants completed both pre- and post-surveys; no primary outcomes were missing.

### Baseline KAP distribution

At baseline, participants exhibited varying levels of knowledge, attitudes, and practices (KAP) concerning intestinal parasitic infections (IPIs). Table 1 summarises the descriptive statistics for pre-K, pre-A, and pre-P scores on intestinal parasitic infections. Descriptive statistics indicated that pre-intervention knowledge scores (Pre-K) were positively skewed (skewness = 1.49) and platykurtic (kurtosis = 1.92), suggesting a clustering of low scores with a few high outliers. The median score was 9.00, which better represented the central tendency than the mean of 13.60 (SD = 14.98) due to the skewed distribution. This reflects limited baseline awareness among the majority of participants regarding the causes, symptoms, and prevention of IPIs.

**Table 1:** Descriptive statistics for Pre-K, Pre-A, and Pre-P scores on intestinal parasitic infections.

Measure	Pre-K	Pre-A	Pre-P
Mean	13.60	54.89	44.40
Median	9.00	55.00	45.40
Std. Deviation	14.98	22.99	23.55
Skewness	1.49	0.03	-0.07
Kurtosis	1.92	-0.65	-0.12

In contrast, attitudes (Pre-A) towards IPIs followed an approximately normal distribution (skewness = 0.03; kurtosis = -0.65). The mean attitude score was 54.89 (SD = 22.99), with a median of 55.00, indicating a relatively balanced spread of perceptions ranging from indifference to concern about IPI prevention and hygiene behaviours. Practices (Pre-P) related to hygiene and infection control also showed a near-normal distribution (skewness = -0.07; kurtosis = -0.12), with a mean of 44.40 (SD = 23.55) and a median of 45.40. While these findings suggest some degree of positive hygiene behaviour, the variability highlights inconsistent practice adherence across the sample. Visual inspection of histograms and Q–Q plots, along with skewness and kurtosis values, supported parametric tests.

### Gender-based comparisons of KAP

Gender differences in KAP scores were observed, but they were not statistically significant. Table 2 shows the mean and standard deviation of knowledge, attitudes, and practices (KAP) scores by gender. Male participants scored higher in knowledge (M = 16.59, SD = 16.18) and attitudes (M = 60.31, SD = 19.92) than females (Pre-K: M = 11.95, SD = 14.14; Pre-A: M = 51.90, SD = 24.17). Conversely, female participants reported better hygiene and preventive behaviours, with a higher mean practice score (M = 45.73, SD = 26.16) than males (M = 41.99, SD = 18.01). However, independent samples t-tests confirmed that these differences were not statistically significant across all three domains, indicating that gender was not a major determinant of baseline KAP levels in this sample. Observed mean differences were small, and 95% confidence intervals spanned zero, suggesting limited practical impact in this sample.

**Table 2:** Mean and standard Deviation of Knowledge, Attitudes, and Practices (KAP) scores by gender.

Measure	Factor	n	Mean	SD	p-value
Pre-K	Male	32	16.59	16.18	0.160
	Female	58	11.95	14.14	
Pre-A	Male	32	60.31	19.92	0.097
	Female	58	51.90	24.17	
Pre-P	Male	32	41.99	18.01	0.474
	Female	58	45.73	26.16	

SD = standard deviation

### Age group variations

Age emerged as a more critical factor in shaping KAP levels. Adolescents (13–17 years) consistently outperformed both children (7–12 years) and adults (≥18 years) across all three domains. Adolescents had the highest mean scores for knowledge (M = 22.50), attitudes (M = 67.33), and practices (M = 53.29). In comparison, children reported scores of M = 10.50 (Pre-K), M = 48.33 (Pre-A), and M = 40.24 (Pre-P), while adults recorded the lowest scores across domains.

Table 3 shows the One-Way ANOVA results for differences in knowledge, attitude, and practice scores across age groups: children, adolescents and adults. One-way ANOVA revealed statistically significant differences across age groups for all three measures: knowledge (F(2,87) = 9.81, p < 0.001), attitudes (F(2,87) = 7.57, p < 0.001), and practices (F(2,87) = 3.38, p = 0.04). Post hoc tests (Student-Newman-Keuls, Tukey HSD, Scheffé) confirmed that adolescents scored significantly higher than both children and adults, who did not differ significantly from each other. This trend suggests that adolescents, potentially due to more recent school-based exposure to health education, demonstrated better baseline readiness for IPI prevention interventions.

**Table 3:** ANOVA for differences in KAP scores across age groups.

		SS	*df*	MS	*F*	*p-*value
Pre-K	Between Groups	3673.80	2	1836.90	9.81	**<0.001**
	Within Groups	16291.80	87	187.26		
	Total	19965.60	89			
Pre-A	Between Groups	6975.56	2	3487.78	7.57	**< 0.001**
	Within Groups	40073.33	87	460.61		
	Total	47048.89	89			
Pre-P	Between Groups	3558.80	2	1779.40	3.38	**0.04**
	Within Groups	45783.20	87	526.24		
	Total	49342.01	89			

SS = Sum of squares, df = degree of freedom, MS = mean of squares,

### Comparison of pre- and post-test scores

Table 4 presents paired-samples statistics for pre- and post-test scores on knowledge, attitudes, and practices related to intestinal parasitic infections among public housing residents in the Klang Valley. Paired samples statistics were computed to examine the differences between pre- and post-test scores across three constructs: Knowledge (K), Attitude (A), and Practice (P). Each construct showed a marked increase from pre- to post-intervention.

**Table 4:** Paired samples t-Test; pre- and post-intervention differences in KAP.

Pair	Measure	Mean	N	SD	SE
1	Post-K	74.12	90	19.57	2.06
	Pre-K	13.60	90	14.98	1.58
2	Post-A	81.78	90	24.89	2.62
	Pre-A	54.89	90	22.99	2.42
3	Post-P	73.72	90	26.77	2.82
	Pre-P	44.40	90	23.55	2.48

SD= Standard deviation, SE= Standard error

For Pair 1 (Knowledge), the mean post-test score (M = 74.12, SD = 19.57) was significantly higher than the mean pre-test score (M = 13.60, SD = 14.98), with a mean difference of 60.52. For Pair 2 (Attitude), participants had a higher post-test mean score (M = 81.78, SD = 24.89) than the pre-test (M = 54.89, SD = 22.99), resulting in a mean difference of 26.89. For Pair 3 (Practice), the post-test mean score (M = 73.72, SD = 26.77) was also higher than the pre-test mean score (M = 44.40, SD = 23.55), yielding a mean difference of 29.32.

### Paired samples correlation analysis

Paired samples correlation analyses revealed no significant relationships between pre- and post-intervention scores for knowledge (r = -0.007, p = 0.949), attitude (r = 0.081, p = 0.449), or practice (r = 0.123, p = 0.249). These findings suggest that initial KAP levels did not predict post-intervention outcomes, emphasising the intervention’s equitable impact across all baseline levels. Regardless of participants’ initial levels of knowledge, attitudes, or practices, the multi-component interactive intervention yielded consistent improvements. The results are presented in Table 5.

**Table 5:** Paired samples correlations between pre- and post-intervention scores on knowledge, attitudes, and practices related to intestinal parasitic infection.

Pair	Measure	N	Correlation	p-value
1	Post-K & pre-K	90	-0.007	0.949
2	Post-A & pre-A	90	0.081	0.449
3	Post-P & pre-P	90	0.123	0.249

### Effectiveness of gamification on knowledge, attitudes, and practices

Following the multi-component interactive intervention, significant improvements were observed across all three KAP dimensions. Paired sample t-tests confirmed that post-intervention scores were significantly higher than pre-intervention scores for knowledge, attitudes, and practices. The results are presented in Table 6. Knowledge improved dramatically from M = 13.60 (SD = 14.98) to M = 74.12 (SD = 19.57), a mean increase of 60.52 points (t(89) = 23.22, p < 0.001). Attitudes shifted positively, increasing from M = 54.89 (SD = 22.99) to M = 81.78 (SD = 24.89), reflecting a mean gain of 26.89 points (t(89) = 7.85, p < 0.001). Practices also improved substantially, with scores rising from M = 44.40 (SD = 23.55) to M = 73.72 (SD = 26.77), amounting to a mean gain of 29.32 points (t(89) = 8.32, p < 0.001).

**Table 6:** Pre- and Post-intervention differences in KAP related to IPIs.

		Paired Differences						
		Mean	SD	SE	95% CI	*t*	*df*	*p*
Pair 1	postK - preK	60.52	24.72	2.61	55.34 - 65.70	23.22	89	<0.001
Pair 2	postA - preA	26.89	32.49	3.42	20.08 - 33.69	7.85	89	<0.001
Pair 3	postP - preP	29.32	33.41	3.52	22.32 - 36.31	8.32	89	<0.001

These results underscore the strong effectiveness of multi-component interactive intervention approaches in promoting health behaviour change and awareness, particularly within underserved urban populations.

## Discussion

This study assessed baseline knowledge, attitudes, and practices (KAP) regarding intestinal parasitic infections (IPIs) among residents of one public housing programme (PHP) in the Klang Valley, Malaysia, and evaluated a multi-component interactive intervention. Pre-intervention findings showed limited knowledge (median = 9.00; skewness = 1.49), despite relatively balanced attitudes (M = 54.89, SD = 22.99) and practices (M = 44.40, SD = 23.55). These findings align with global patterns of low IPIs awareness among vulnerable groups [10,13,20] and reflect Malaysian trends, particularly among rural and indigenous communities [21,22]. The structural and environmental conditions observed in this PHP—overcrowding, shared sanitation, and inadequate waste management—are not unique to Malaysia and are common in rapidly growing cities worldwide, such as Mumbai, Nairobi, and Rio de Janeiro [15–17], suggesting that lessons from this community may be relevant to other urban low-income settings.

Sociodemographic factors shaped baseline KAP outcomes. Adolescents (13–17 years) outperformed both children and adults in all KAP domains, which may reflect more recent exposure to school-based hygiene education and deworming initiatives [23]. Adults showed the weakest outcomes, indicating the need for community-level interventions that extend beyond formal education. Gender analysis revealed no statistically significant differences, although males had slightly higher knowledge (M = 16.59) and attitudes (M = 60.31), while females scored slightly better in practices (M = 45.73), possibly reflecting household responsibilities and behavioural patterns. These differences highlight the importance of age-appropriate and role-sensitive tailoring of content.

The multi-component interactive intervention led to statistically significant improvements across all domains: knowledge (+60.52, t(89) = 23.22, p < 0.001), attitudes (+26.89, t(89) = 7.85, p < 0.001), and practices (+29.32, t(89) = 8.32, p < 0.001). These results support effectiveness of a multi-component interactive intervention that includes a game-based participatory learning approach in enhancing public health education, consistent with evidence from comparable interventions, such as “Worms and Ladders” in Nigeria [24] and the “Magic Glasses” programme in China and the Philippines [25]. Although we did not compute standardised effects here, the magnitude of mean changes indicates large gains in knowledge and moderate-to-large gains in attitudes and practices, which is congruent with the KAP framework, wherein knowledge acquisition can catalyse attitudinal shifts and downstream behaviour change [26]. As soil-transmitted helminths and other IPIs are sustained at the interface between people, animals, and the urban environment, a behaviour-focused intervention that strengthens hygiene and environmental practices naturally fits within a One Health approach to interrupting enteric parasite transmission.

Participant engagement was high throughout the sessions, and the culturally adapted, low-cost activities increased accessibility and learning retention. Residents responded enthusiastically to content presented through interactive games, group-based challenges, and visual aids, suggesting a strong preference for health education that is inclusive, engaging, and free from jargon.

The intervention created a supportive learning environment in which participants could explore, discuss, and make mistakes without judgement, in contrast to the didactic and often inaccessible nature of traditional health talks and outreach programmes. The strength of this approach lies in connecting knowledge to action using real-life examples and demonstrations (e.g., microscope viewing). The observed improvements across all KAP domains are consistent with the KAP framework, whereby increased knowledge may contribute to more favourable attitudes and healthier practices. Together, these findings support the use of a multi-component interactive intervention to enhance community health education on intestinal parasitic infections.

Low-income urban communities often lack access to consistent health messaging. Many participants, especially adults, shared that this was their first time learning about IPIs in such an accessible way. Just as residents wanted more affordable, well-connected neighbourhoods, our study’s participants voiced the need for public health education that reaches all strata of the community. This study affirms that vulnerable communities are willing and able to engage meaningfully in health education when approached with respect, creativity, and cultural relevance.

These findings also carry broader implications for public health services. The intervention’s low-cost, cultural relevance, and scalability suggest strong potential for adaptation in other marginalised communities. By tailoring activities to diverse age groups, the study promotes inclusive health learning and offers practical implications for NGOs, public health practitioners, and policymakers. In doing so, it supports the United Nations Sustainable Development Goals (SDGs), particularly SDG 3 (Good Health and Well-being) by promoting disease prevention, SDG 4 (Quality Education) by creating accessible, lifelong learning opportunities for health, and SDG 10 (Reduced Inequalities) by empowering communities across gender and age groups [27].

At the same time, this study cements the fact that behavioural change interventions alone cannot overcome persistent environmental and infrastructural shortcomings; rather, they must operate in tandem with systemic improvements to actively reduce transmission and sustain long-term health benefits. In similar settings, such multi-component interactive interventions could be coupled with improvements in local water, sanitation, and animal waste management to create a more comprehensive One Health package for urban public housing communities.

Future research should examine the long-term impact of the intervention on health behaviours and explore its applicability across different neglected tropical diseases (NTDs) and delivery formats. Such efforts would support the development of scalable, inclusive strategies to improve health literacy among marginalised populations.

While numerous studies emphasise the cognitive benefits of gamification and multi-component interactive interventions in health education, less is known about how vulnerable urban populations perceive and respond to such interventions. This study, situated within a high-risk, low-income setting in Kuala Lumpur, provides insights not only into the measurable outcomes of such intervention on intestinal parasitic infections (IPIs) but also the underlying aspirations and expectations of underserved communities regarding public health education. The social and environmental drivers of IPIs described here, crowded housing, shared facilities, and contaminated play spaces, are common features of many low-income neighbourhoods, which suggests that this approach could be adapted to other urban One Health hotspots beyond Kuala Lumpur.

Despite promising outcomes, this study has several limitations. The sample was restricted to a single PHP community, which may limit the generalisability of findings to other low-income populations with differing socio-cultural contexts. At the same time, many of the structural risk factors we observed, such as overcrowding, unstable WASH services, and informal contact with contaminated environments, are widely reported in other rapidly urbanising areas, which supports cautious adaptation of this approach to similar settings. The reliance on self-reported KAP data introduces potential response biases, including social desirability bias. Additionally, the intervention’s impact was assessed immediately post-implementation, limiting insight into long-term knowledge retention or behavioural change. Variability in participant engagement, particularly among adults, also suggests that the gamification approach may not be equally effective across all age groups or learning preferences. These limitations should be considered when interpreting the results and planning future studies.

## Conclusion

This multi-component interactive intervention was effective in enhancing knowledge, attitudes, and practices (KAP) related to intestinal parasitic infections (IPIs) among residents of a public housing programme (PHP) in the Klang Valley, Malaysia. After the intervention, participants’ knowledge increased significantly by 5.45 times, attitudes improved by 1.49 times, and practices increased by 1.66 times compared to their baseline scores. Given that similar social and environmental conditions exist in low-income housing in many cities, this type of health education could be incorporated into broader One Health strategies to reduce enteric infections in dense urban communities.

The insights from this study also emphasise the importance of tailored health education strategies based on age and gender to improve overall community health outcomes, enhance health literacy, and promote behavioural change. The finding also reinforces that gamification and multi-component interactive intervention are effective, age-inclusive, and culturally adaptable tools for health education, particularly in underserved, high-risk communities, and proves to be a promising strategy for future interventions.

It is recommended that similar multi-component interactive programmes be implemented and evaluated in broader populations to further validate these results. The findings of this study not only contribute to the academic literature but also provide practical insights for health educators, policymakers, and community health workers aiming to develop more effective health interventions for marginalised populations.
